# Modulation of Apoptosis by Plant Polysaccharides for Exerting Anti-Cancer Effects: A Review

**DOI:** 10.3389/fphar.2020.00792

**Published:** 2020-05-27

**Authors:** Qing-xia Gan, Jin Wang, Ju Hu, Guan-hua Lou, Hai-jun Xiong, Cheng-yi Peng, Qin-wan Huang

**Affiliations:** College of Pharmacy, Chengdu University of Traditional Chinese Medicine, Chengdu, China

**Keywords:** plant polysaccharides, apoptosis, cancer, pathway, natural products

## Abstract

Cancer has become a significant public health problem with high disease burden and mortality. At present, radiotherapy and chemotherapy are the main means of treating cancer, but they have shown serious safety problems. The severity of this problem has caused further attention and research on effective and safe cancer treatment methods. Polysaccharides are natural products with anti-cancer activity that are widely present in a lot of plants, and many studies have found that inducing apoptosis of cancer cells is one of their important mechanisms. Therefore, this article reviews the various ways in which plant polysaccharides promote apoptosis of cancer cells. The major apoptotic pathways involved include the mitochondrial pathway, the death receptor pathway, and their upstream signal transduction such as MAPK pathway, PI3K/AKT pathway, and NF-κB pathway. Moreover, the paper has also been focused on the absorption and toxicity of plant polysaccharides with reference to extant literature, making the research more scientific and comprehensive. It is hoped that this review could provide some directions for the future development of plant polysaccharides as anticancer drugs in pharmacological experiments and clinical researches.

## Introduction

Cancer is one of the most life-threatening diseases in the world (Zarei et al., 2016). In 2018, it has caused more than 9.6 million deaths as reported by the World Health Organization (WHO). The number of cancer patients continues to grow globally, despite the ongoing development of modern medical methods for preventing disease (López-Gómez et al., 2013; Surbone and Halpern, 2016; Srivastava et al., 2019). It is well known that the treatment of cancer is expensive and time-consuming. The result is that cancer places a huge burden on individuals, families, communities, and health systems, and causes tremendous harm in physical, emotional, and financial of patients (Veenstra et al., 2014; Li Y. et al., 2018; Saad et al., 2019).

The occurrence of cancer is mainly caused by a series of changes in the genome and epigenome (Shen and Laird, 2013). These changes cause the cells to continuously proliferate and escape apoptosis, thereby disrupting the homeostasis of the tissue (Khan et al., 2016). It has been found that promoting cancer cell apoptosis is one of the effective methods to treat cancer (Mortezaee et al., 2019b). That is also the main mechanism of chemo(radio)therapy which is the most common cancer treatment (Agool et al., 2011; Deng et al., 2012; Mortezaee et al., 2019a). However, most of these drugs will cause normal cells apoptosis and lead to serious physical damage (Cheki et al., 2018; Zhang H. et al., 2018), such as myelosuppression, cardiotoxicity, hepatotoxicity, nephrotoxicity, and gastrointestinal toxicity (Liu et al., 2018; Oun et al., 2018; Zhang Q. et al., 2018). Finding safe and effective treatments has been a long-term goal of improving cancer.

**Figure 1:**
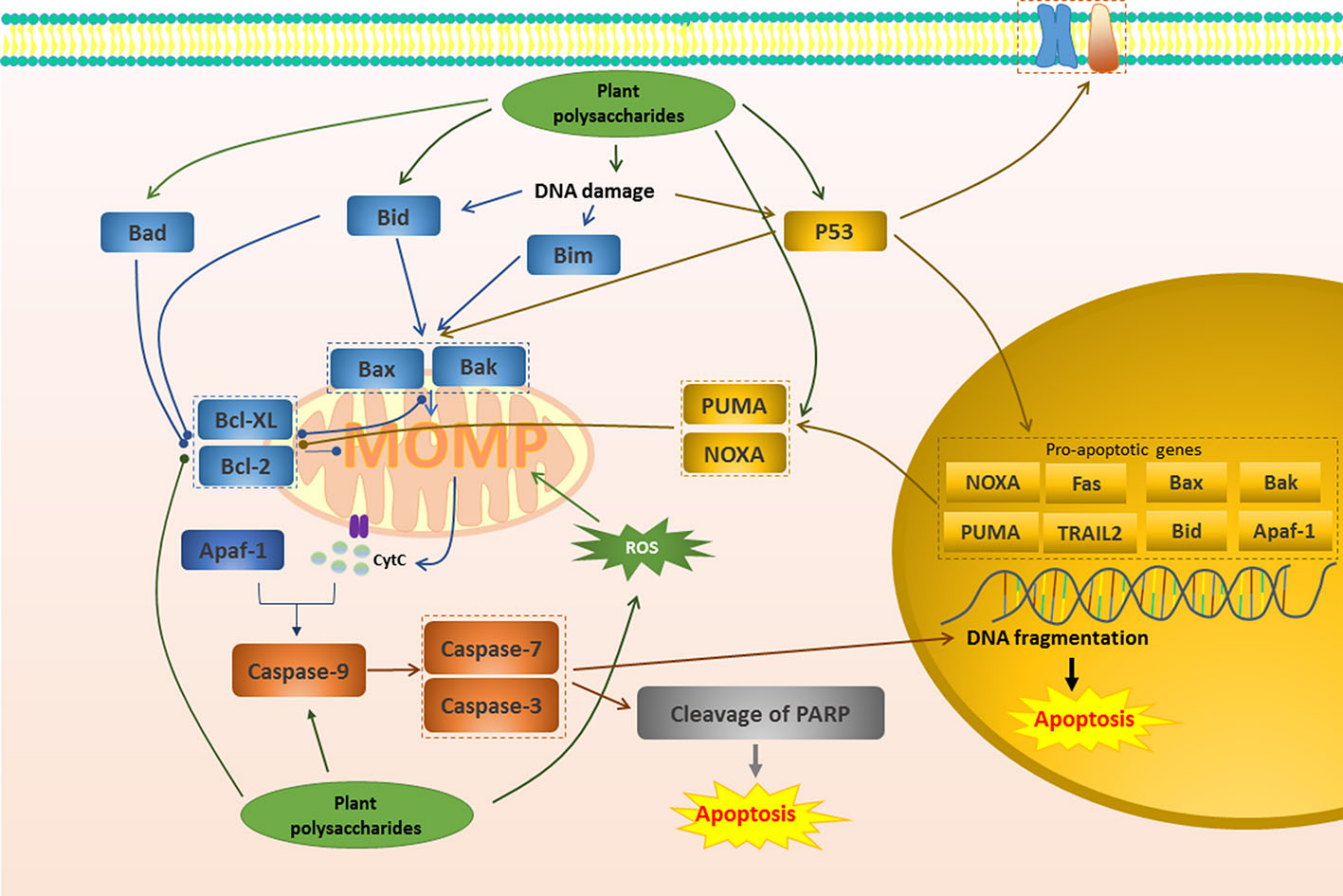
Mitochondrial apoptotic pathway in cancer induced by plant polysaccharides. 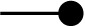 with different colors indicate inhibition/reduction, 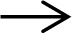 and 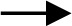 with different colors indicate increase/promotion.

**Figure 2:**
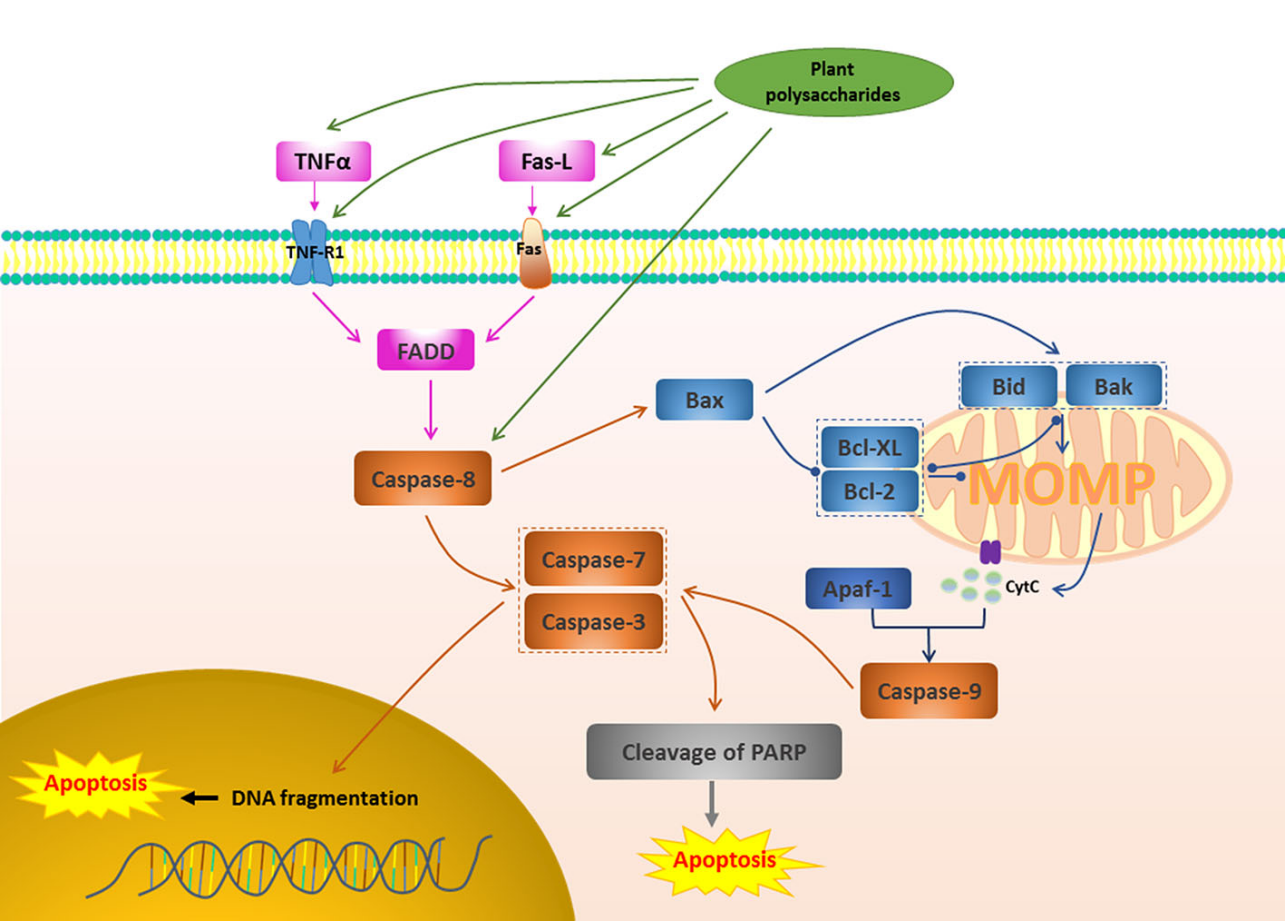
Death receptor apoptotic pathway in cancer induced by plant polysaccharides. 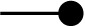 with different colors indicate inhibition/reduction, 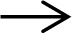 and 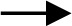 with different colors indicate increase/promotion.

**Figure 3:**
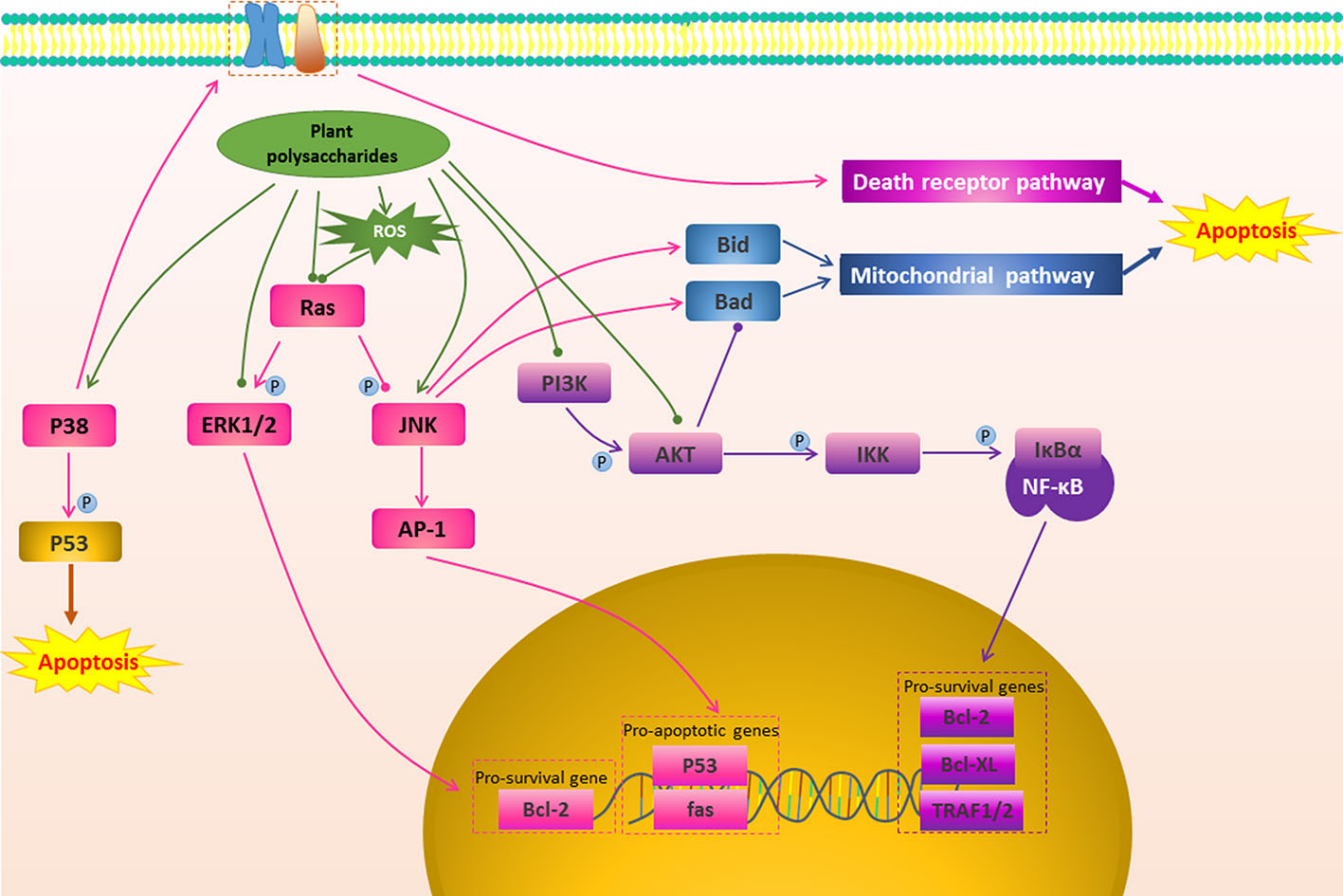
The regulation of other apoptosis signal conduction in cancer by plant polysaccharides 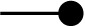 with different colors indicate inhibition/reduction, 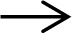 and 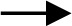 with different colors indicate increase/promotion.

Natural products isolated from plants are gradually recognized for their high efficiency and safety (Bishayee and Sethi, 2016). In cancer treatment, plant extracts have been widely used (Sun et al., 2019; Zabaiou et al., 2019). Polysaccharide is one of the active ingredients in a lot of plants, and shows low toxicity and high efficiency in the treatment of cancer (Jiao et al., 2016; Khan et al., 2019). Such as plant polysaccharides from genus Astragalus, Ginseng, Schisandra, and many others have been shown to be selective for the cytotoxicity of tumor cells, so they can kill cancer cells without the usually associated side effects. And the detailed molecular mechanism mainly involves the inhibition of cancer cell proliferation through promoting apoptosis. In recent years, studies have found that a variety of plant polysaccharides can regulate cancer cells apoptosis *in vivo* and *in vitro*. However, no systematic studies have evaluated the ability of plant polysaccharides to induce apoptosis. Therefore, this article reviews plant polysaccharides by inducing cancer cells apoptosis *via* multiple pathways and multiple targets to treat cancer, as shown in **Figures 1**–**3** and **Table 1**, hoping to provide a reference for the treatment of cancer by plant polysaccharides in subsequent studies.

**Table 1:** Effects of plant polysaccharides on apoptosis in cancer.

Drugs	Monosaccharide composition/structure	Model	Dose and treat time	Activity and type of cancer	Apoptotic pathway	Involved mechanism	Ref
*Andrographis paniculata* (Burm.f.) Nees polysaccharide (APWP)	Galatose, arabinose, rhamnose (6:3:1)	HepG2 cells.	0–1,000μg/ml, 24, 48, and 72 h	AR: 65.1% CV: 34.7% Hepatocellular carcinoma	Mitochondrial Pathway	Bax↑; MMP↓; Cytochrome c↑; Caspase-9, Caspase-3↑; annexin V-positive↑	(Zou et al., 2015)
*Asparagus officinalis L*. polysaccharide (AP)		MSC-2cells	0.25, and 0.5mg/ml, 72 h	AR: 12.1% CV: About 50% Colon cancer	Mitochondrial Pathway	Bax, Caspase 9↑; Bcl-2↓; P-NF-κB p65 ↑; TLR4↑	(Zhang W. et al., 2018)
*Artemisia capillaris* Thunb polysaccharide (WACP)	Arabinose, galactose (4:2)	CNE-2 cells	0–200μg/ml, 24, 48, 72 h	AR: 47.65% Nasopharyngeal carcinoma	Mitochondrial Pathway	MMP↓; Cytochrome c↑; Caspase-3/9↑	(Feng et al., 2013)
*Astragalus mongholicus* Bunge (Astragali Radix) Polysaccharides	Rhamnose, arabinose, glucose, galactose, Glucuronic acid (0.03:1.00:0.27:0.36:0.30)	SW620 cells	0. 1-1. 0 g/L, 48 h	AR: 37. 63% Colon cancer	Mitochondrial Pathway	Bax/Bcl-2↑; Caspase-3, Caspase-9, Cytochrome c ↑	(Yan et al., 2017)
A*stragalus mongholicus* Bunge polysaccharide (APS)		Mice injected with MDA-MB-231 cells	Orally, 200, 400 mg/kg for 21 days	AR: 62. 87% TIR: 57. 57% Breast Cancer	Mitochondrial Pathway	Bcl-2↓; Bax, Caspase-9, Caspase-7↑	(Xie R.-D. et al., 2019)
A*stragalus mongholicus* Bunge polysaccharide (APS)		CNE-1, CNE-2, and SUNE-1	10–80μg/ml, 48 h	AR: Over 40% TIR: Over 75% CV: Under 50% Nasopharyngeal Carcinoma	Mitochondrial Pathway	Bcl-2↓; Bax, Caspase-3, Caspase-9↑	(Zhou Z. et al., 2017)
		Mice injected with CNE-2 cells	40mg/kg (i.p.) twice a week for 4 weeks				
*Astragalus mongholicus* Bunge cold-water-soluble polysaccharide (APS4)	Glucose, rhamnose, arabinose, xylose, mannose, galactose (12.1: 0.3: 0.6: 1.0:1.0: 1.7.)	MGC-803 cells	0–800μg/ml, 24 h	AR: 20.60% CV: 54.23% Gastric carcinoma	Mitochondrial Pathway	Bax/Bcl-2↑; Cytochrome c↑; Caspase-9/-3↑; PARP↓; ROS↑	(Yu et al., 2019)
*Aralia elata* (Miq.) Seem. polysaccharide (AEP-1)	Glucose, galactose, arabinose (6.0: 3.0: 1.0)	U-2 OS cell	25, 50, and 100μg/ml, 24, 48, and 72 h	CV: About 50% Osteosarcoma	Mitochondrial Pathway	DNA fragmentation; Bax/Bcl-2↑; MMP↓; Cytochrome c↑; Caspase-9 and Caspase-3↑; PAPR↓	(Liu et al., 2016b)
*Atractylodes macrocephala* Koidz. polysaccharide (AMPs)		C6 cells	50, 100, and 200μg/ml, 48 h	CV: 19.1% Glioma	Mitochondrial Pathway	MMP↓; Cytochrome c↑; Capase-3, Caspase-9↓; PARP↓	(Li X. et al., 2014)
*Camellia sinensis* polysaccharide (GTP)	Glucose	PC cells	25–100μg/ml, 48 h	AR: 73.2% CV: About 50% Prostate cancer	Mitochondrial Pathway	Bax/Bcl-2↑; Caspae-3↑; miR-93↓	(Yang et al., 2019)
*Citrus × aurantiifolia* (Christm.) Swingle polysaccharide (CAs)	Rhamnose, arabinose, galactose, glucose, mannose, galacturonic acid (0.67: 7.67: 10.83: 3.83: 4.00: 1.00)	Mice injected with H22 cells	Orally, 50 and 250mg/kg for 3 weeks	TIR: 58.85% Hepatocellular carcinoma	Mitochondrial Pathway	Bcl-XL, Mcl-1↓; Caspase 3↑	(Zhao Y. et al., 2017)
*Curcuma kwangsiensis* S.G.Lee & C.F.Liang polysaccharides (CKP)	Fructose, xylose, mannose, glucose, galacose (25.0, 25.0, 10.0,12.5,12.5)	CNE-2 cells	12.5–100.0μg/ml, 48 h	AR: 42.85% CV: 76.52% Nasopharyngeal	Mitochondrial Pathway	Bcl-2↓; p53↑	(Zeng et al., 2012)
*Cymbopogon citratus* (DC.) Stapf polysaccharides (F1 and F2)	F1; Glucose, Galactose, Xylose, Mannose (5.1,11.5,74.5,3.2) F2: Glucose, Galactose, Xylose, Mannose (6.2, 9.8, 69.5, 7.3)	Siha and LNCap cells	100–1,000μg/mL, 24, 48 and 72 h	CV: Under 60% Reproductive cancer	Mitochondrial Pathway	COX-2 ↓; DNA fragmentation; MMP ↓; p53 expression↑; Caspase 3↑; Bcl-2 family genes↓; Cytochrome c↑	(Thangam et al., 2014)
*Glycyrrhiza inflata* Batalin polysaccharide (GIAP1)	Glucose, galactose, and mannosein (2.3:1.2:1.2)	SCC-25 cells	50, 100, and 200μg/mL, 48 h	AR:64.07% Oral cancer	Mitochondrial Pathway	Bax/Bcl-2↓; MMP↓; Cytochrome c↑; Capase-3 and Caspase-9↑; PARP↓	(Zeng et al., 2015)
*Scleromitrion diffusum* (Willd.) R.J.Wang (syn. *Hedyotis diffusa* Willd) polysaccharide (SDP)	Glucose, galactose, mannose (2.0: 1.0: 1.0)	A549 cells	25, 100, and 200μg/ml, 48 h	AR:66.08% TIR: Nearly 50% Lung cancer	Mitochondrial Pathway	Caspase-9 and -3↑; Cytochrome c↑; Bax↑	(Lin et al., 2019)
		Mice injected with A549 cells	50, 100 mg/kg for 15 days				
*Scleromitrion diffusum* (Willd.) R.J.Wang (syn. *Hedyotis diffusa* Willd) polysaccharides (SDP)	Rhamnose, glucose, galactose, arabinose, mannose (4.31:4.16:4.49:9.22:27.8)	Hep2 cells	25–800μg/ml, 24 h	AR: 67.45% CV: 10.29% Larynx squamous carcinoma	Mitochondrial Pathway	Caspase-3, Caspase-8, Caspase-9↑; Bcl-2↓	(Wu C. et al., 2017)
*Palisada perforata* (Bory) K.W.Nam (syn. *Laurencia papillosa* (C.Agardh) Greville) polysaccharide (ASPE)		MDA-MB-231 cells	5–100 µg/ml, 24 h	AR: 50 % CV: About 20% Breast cancer.	Mitochondrial Pathway	Cip1/p21, Kip1/p27↑; cyclins D1, D2, E1 transcripts, Cdk2, Cdk4, Cdk6↓; Bax transcripts↑; Bcl-2↓; Caspase-3↑; ROS↑	(Murad et al., 2016)
*Paeonia × suffruticosa* Andrews (Peony seed dreg) polysaccharide (CASS)	L-galactose, L-arabinose, D-glucose (34.43, 26.39, 21.80)	Pc-3 cells HCT-116 cells MCF-7 cells Hela cells	50–500 µg/ml, 48 h	AR: 20.22% (Pc-3), 17.87% (HCT-116), 30.94% (Hela), 38.73% (MCF-7) Prostate cancer Colon cancer Breast cancer Cervical cancer	Mitochondrial Pathway	mRNA Cyclin A/B1/D1/E1↓; CDK-1/2/4/6, p15/16/21/27↓; p53↑; Survivin, Bcl-2↓; Caspase-3, Caspases-8 and Caspases-9↑; Cytochrome c, Bax↑; Bcl-2↓; PARP↓	(Zhang F. et al., 2017)
*Punica granatum* L. (Pomegranate) peels polysaccharide (PPP)	Total sugar, uronic acid, and protein (72.4, 19.5, 9.7)	U-2 OS cells	0–400μg/ml, 24–96 h	AR: 49.73% CV: Over 40% Osteosarcoma cancer	Mitochondrial Pathway	Bax/Bcl-2↑; MMP↓; Cytochrome c↑; Caspase-9 and Caspase-3↑; PARP↓	(Li J. et al., 2014)
*Portulaca oleracea L*. polysaccharide (POL-P3b)	Glucose, galactose (0.75:1.00)	HeLa cell	50–1,000μg/mL, 24, 48, and 72 h	TIR: 50.28% Cervical carcinoma	Mitochondrial Pathway	Cytochrome c↑; Caspase-9, Caspase-3↑; wild-type p53↑	(Zhao et al., 2013)
*Cucurbita pepo* L. (Pumpkin) polysaccharide (PPW)	Galactose, mannose, glucose, arabinose (2.02:2.05:1.00:0.52)	HepG2 cells	100, 200, and 400μg/ml, 24, 48, and 72h	AR: 70.3% CV: Over 30% Hepatocellular carcinoma	Mitochondrial Pathway	Bax↑; Bcl-2↓; Caspase-9, Caspase-3↑; PARP↓	(Shen et al., 2016)
*Pinellia ternata* (Thunb.) Makino polysaccharide (PTPA)		Cholangiocarcinoma cell lines	25–200μg/ml, 24 h	AR: 78.9% CV: Under 50% Cholangiocarcinoma	Mitochondrial Pathway	Bax/Bcl-2↑; Caspase-9 and Caspase-3↑; 67LR, Cdc42↓	(Li Y. et al., 2016)
*Carthamus tinctorius* L. (Safflower) polysaccharide (SPS)		MCF cells	0.04, 0.08, 0.17, 0.34, 0.68, 1.36mg/ml, 48 h	AR: 54.5% TIR: Over 80% Breast cancer	Mitochondrial Pathway	Bcl-2↓; MMP-9 ↓; TIMP 1↓	(Luo et al., 2015)
*Carthamus tinctorius* L. (Safflower) polysaccharide (SPS)		HN-6 cells	0-1.28 mg/mL, 24–72 h	TIR: About 50% Tongue squamous cell carcinoma	Mitochondrial Pathway	Bcl-2, COX-2↓; Bax, cleaved Caspase-3↑	(Zhou et al., 2018)
*Sanguisorba officinalis* L. (Sanguisorbae radix) polysaccharide polysaccharide (SRP)		HL-60 cells	50, 100, 150, 200, and 400μg/ml, 24 h	AR: 70.2% CV: Under 50% Leukemia	Mitochondrial Pathway	MMP↓; Cytochrome c↑; Bcl-2 protein and mRNA↓; Bax↑; Caspase-9, Caspase-3↑	(Wu et al., 2014)
*Sargassum wightii* Greville ex J.Agardh polysaccharides (SWP)	Galactofuranose, arabinose (64.6:11.2)	MCF7 and MDA-MB-231 cells	0–500μg/ml, 24 h	Breast cancer	Mitochondrial Pathway	ROS↑; Nuclei damage; Caspase3/9↑	(Vaikundamoorthy et al., 2018)
*Sargassum integerrimum* Tseng & Lu polysaccharide (SPSa)		A549 cells	0–1.5 mg/ml for 12 and 24 h	AR: 28.8% Lung cancer	Mitochondrial Pathway G2/M phase arrest	MMP↓; ROS↑; P53, Bax↑; Bcl-2↓; Caspase-3, Caspase-9↑; PARP↓; G2/M phase↓	(Liu et al., 2016a)
		Transgenic zebrafish	1, 4mg/ml for 48 h				
Se-containing polysaccharides from *Ginkgo biloba L*. leaves (Se-GBLP)	93.7% carbohydrate	T24 cells	50–200μg/ml, 24, 48 or 72 h	AR: 70.3% CV: 30.2% Bladder cancer	Mitochondrial Pathway	Bax↑; Bcl-2↓; MMP↓; Caspase-9, Caspase-3↑; PARP↓	(Chen et al., 2017)
*Pyracantha fortuneana* (Maximowicz) H. L. Li, J. Arnold Arbor. (Se-PFPs)	Carbohydrate (93.7%), uronic acid (2.1%), and 3.7μg/g of Se	HEY and SKOV3 cells	0–1,000μg/ml, 24 h	AR: 41.4% (HEY) AR: 30.4% (SKOV3) TIR: Over 40% Ovarian cancer	Mitochondrial Pathway	cyclin D1, Bcl-2, MMP-9↓; PARP↓; Caspase-3 and Caspase-9↑; β-catenin↓; p-β-catenin↑	(Sun et al., 2016)
		Female mice injected with HEY cells	Orally, 400 mg/kg for 28 days				
*Millettia pulchra* (Voigt) Kurz (Yulangsan) polysaccharide (YLSPS)		4T1 cells	10% medicated serum, 24h	AR: over 30% TIR: over 35% Breast Cancer	Mitochondrial Pathway	Bcl-2↓; Bax↑; Bcl-2/Bax↓; Cytochrome c↑; Caspase-3↑	(Qin et al., 2019)
		Mice injected with 4T1 cells	150, 300, 600 mg/kg for 7 days				
*Boschniakia rossica* (Cham. & Schltdl.) B.Fedtsch. polysaccharide (BRP)		Hep2 cells	50, 100, 200, 400 mg/L,48h	AR:30.9% Laryngeal cancer	Mitochondrial Pathway Death receptors Pathway	Caspase-3↑; p53↑; Bcl-2/Bax↓; NO↑; TNF↑	(Yao et al., 2017)
*Boschniakia rossica* (Cham. & Schltdl.) B.Fedtsch. Polysaccharide (BRP)		Hep2 cells	100, 200, and 400 ug/ml, 24 h	AR: 60% Laryngeal carcinoma	Mitochondrial Pathway Death receptors Pathway	Cleavage of pro-Caspase-3, pro-Caspase-8, and pro-Caspase-9↑; DR5, Bax↑; Bcl-2↓	(Wang et al., 2014)
*Zingiber officinale* Roscoe (Ginger) polysaccharide (GP)	L-rhamnose, D- arabinose, D-mannose, D-glucose, D-galactose (3.64: 5.37: 3.04: 61.03: 26.91)	HepG2 cells	0–0.4 mg/ml, 12 h	AR: 16.84% CV: 50.37% Hepatocellular carcinoma	Mitochondrial Pathway Death receptors Pathway	Bax, Fas, FasL↑; Caspase-3, p21, p53↑; Bcl-2↓	(Wang et al., 2019)
*Lycium barbarum* L. polysaccharide (LBP)		MLL - ALL cells	100, 500, 1,000mg/l, 12 h	AR: Over 25% Leukemia	Mitochondrial Pathway Death receptors Pathway	DR5↑; Caspase-8, Bid, Caspase-3, Caspase-9, Bad↑; Bcl-2↓	(Chen C. et al., 2019)
*Astragalus mongholicus* Bunge polysaccharide (APS)	89.75% total carbohydrate and 9.3%uronic acid	MCF-7 cells	50–1,000μg/mL, 24 h	AR:29.7% Breast cancer	Death receptors Pathway	NO and TNF-α↑; Bax/Bcl-2↑	(Li W. et al., 2019)
*Gracilariopsis lemaneiformis* (Bory de Saint-Vincent) E.Y.Dawson, Acleto & Foldvik Polysaccharide (PGL)	D-galactose and 3,6-anhydro- L -galactose (57.38:41.2)	A549 cells	0–100 µg/ml, 72 h	AR: Over 5% Lung cancer	Death receptors Pathway	Fas/FasL↑	(Kang et al., 2017)
*Tussilago farfara* L. flower buds polysaccharide (TFPB1)	Rhamnose, galacturonic acid, glucose, galactose, and arabinose (13: 13: 1: 7: 12)	A549 cells	31.2–1,000μg/mL, 48 h	AR:40% Lung cancer	Death receptors Pathway PI3K/AKT Pathway	p-AKT↓; Caspase-3↑; Fas, FasL↑; Bax↑; Bcl-2↓	(Qu et al., 2018)
*Carthamus tinctorius* L. (Safflower) polysaccharide (SPS)		A549 and YTMLC-90 cells	0.04 to 2.56 mg/ml, 24, 48, and 72 h	TIR: About 80% Lung cancer	PI3K/AKT Pathway Death receptors Pathway G2/M phase arrest	Bax mRNA, Caspase-3↑; CDC25B, cyclin B1↓; AKT, p-AKT, PI3K↓; TNF- α, IL-6↑	(L J. Y.i et al., 2016)
		Mice injected with A549 cells	15 to 135 mg/kg (i.p.) for 30 days				
A novel polysaccharide derived from algae		MCF-7 cells	1–1,000 µg/ml, 48 h	AR: Over 16% Breast cancer	MAPK Pathway	P-JNK↑; Phosphorylation and expression of p53↑; Caspase-9 and Caspase-3↑; ROS↑	(Xie et al., 2018)
*Astragalus mongholicus* Bunge polysaccharide (APS)		MG63 cells	1, 5, 10, 20mg/ml, 24 h	AR: About 10% Osteosarcoma	MAPK Pathway (JNK pathway)	miR-133a↑; cyclinD1↓; p21↑; Bcl-2↓; Bax↑; Caspase-9 and Caspase-3↑	(Chu et al., 2018)
*Astragalus mongholicus* Bunge polysaccharide (APS)		SKOV3 cells	50–1,600μg/ml, 24, 48, and 72 h	AR: Over 40% CV: About 60% Ovarian cancer	MAPK Pathway (JNK pathway)	Bcl2↓; Bax, Caspase-3↑; JNK1/2↑	(Li C. et al., 2018)
*Schisandra chinensis* (Turcz.) Baill. polysaccharide (SCP)		RCC cell lines (Caki-1, Caki-2, and ACHN)	25–800μg/ml, 48 h	AR: About 50% CV: Under 50% Renal cell carcinoma	MAPK Pathway Mitochondrial Pathway	MMP↓; Cytochrome c↑; Bax/Bcl-2↑; Caspase-3/9↑; PARP↓; p- ERK1/2↓	(Liu et al., 2014)
*Aconitum coreanum* (H.Lév.) Rapaics polysaccharide (CACP)		H22 cells	10, 20, and 40μg/ml for 48 h	AR: 43.3% TIR: 45.9% Hepatocellular carcinoma	PI3K/AKT Pathway MAPK Pathway	PTTG1 mRNA↓; p-AKT↓; p-p38MARK ↑	(Liang et al., 2015)
*Astragalus mongholicus* Bunge polysaccharide (APS)		ASPC-1, PANC-1 cells	50–400 μg/ml, 24 h	RA: Over 30% (ASPC-1) Over 20% (PANC-1) Pancreatic cancer	MAPK Pathway PI3K/AKT Pathway	P-AKT, p-ERK, MMP-9↓;	(Wu et al., 2018a)
*Panax ginseng* C.A.Mey. polysaccharide (GPS)		MG-63 cell	20, 10, 5, and 1mM, 24 h	AR: 57% Osteosarcoma	MAPK Pathway PI3K/AKT Pathway	P-p38, p-AKT↓; Bax, Caspase3↑; Bcl-2↓	(Zhang X. et al., 2017)
*Gracilariopsis lemaneiformis* (Bory de Saint-Vincent) E.Y.Dawson, Acleto & Foldvik polysaccharide selenium nanoparticles (GLP- SeNPs)		U87 cells	0.2, 1, 5, and 10 mg/ml, 72 h	CV: 44.6% Glioma	MAPK Pathway PI3K/AKT Pathway	P-p53↑; p-p38, p-JNK↑; p-AKT, p-ERK↓	(Jiang et al., 2014)
*Hordeum vulgare L*. polysaccharide (BP-1)	Glucose, xylose, arabinose, rhamnose (8.82:1.92:1.50:1.00)	HT-29 cells	0–150μg/ml, 48 h	AR: 49.17% Colon cancer	MAPK Pathway NF-κB Pathway	P-JNK↑; Ras↓; p-ERK↓; NF-κB↓; Bcl-2↓; Bax↑; Cytochrome c, Caspase-8, Caspase-9↑	(Cheng et al., 2016)
*Astragalus mongholicus* Bunge polysaccharide (APS) and Apatinib		AGS cells	0–600μg/ml, 24h	AR: Over 20% TIR: Over60% Gastric cancer	PI3K/AKT Pathway	p-AKT ↓; MMP-9↓	(Wu et al., 2018b)
*Capsosiphon fulvescens* (C.Agardh) Setchell & N.L.Gardner polysaccharide (Cf-PS)	Xylose, mannose (85:15)	AGS cells	0–5 mg/ml, 24 h	CV: About 40% Gastric cancer	PI3K/AKT Pathway	Bad, Caspase-3↑; Bcl-2↓; p-IGF-IR↓; Akt↓	(Kwon and Nam, 2007)
*Cornus officinalis* Siebold & Zucc. polysaccharide polysaccharide (COP)		HepG2 cells	6.25, 12.5, and 25 mg/ml, 72 h	AR: 20.1% Hepatocellular carcinoma	PI3K/AKT Pathway	Bax, cleaved-Caspase-3, Klotho↑; Ki67, Bcl-2, p-PI3K/PI3K, p-AKT/AKT↓	(Li and Sun, 2019)
*Dipsacus asperoides* C.Y.Cheng & T.M.Aiwater-soluble polysaccharide (ADAPW)	Glucose, rhamnose, arabinose mannose (8.54:1.83:1.04:0.42.)	HOS cells	100, 200, and 400μg/ml, 24 h	AR: 55.3% Osteosarcoma	PI3K/AKT Pathway	MMP↓; ROS↑; PI3K, p-AKT↓	(Chen et al., 2013b)
*Glycyrrhiza inflata* Batalin polysaccharide (GPSa)		Mice injected with H22 cells	Orally, 25, 50, and 75mg/kg for 10 days	TIR: Over 85% Hepatocellular carcinoma	PI3K/AKT Pathway	P53 DNA and protein↑; p-PI3K and p-AKT↓	(Chen et al., 2013a)
		H22 and BEL7402 cells	2–1,250μg/ml, 24, 48 h				
*Scutellaria barbata* D.Don water-soluble polysaccharide (SPS2p)	Arabinose, mannose, glucose, galactose (1.31:1.00:3.59:1.59)	HT29 cells	10, 20, 40, and 80 mg/l, 24 h	AR: Over 30% Colon cancer	PI3K/AKT Pathway	Bax, Bak↑; Bcl-2, FN↓; E-cadherin mRNA↑; N-cadherin, vimentin mRNA, p-AKT/AKT↓	(Sun et al., 2017)
*Aster tataricus* L.f. polysaccharide (ATP-II)	Glucose, galactose, mannose, rhamnose, and arabinose (2.1:5.2:2.1:1.0:1.2)	C6 cells	50–1,000μg/ml, 24, 48, and 72 h	Glioma	PI3K/AKT Pathway Mitochondrial Pathway	DAN damage; Bax/Bcl-2↑; Caspase-3, Caspase-8, Caspase-9↑; AKT↓	(Du et al., 2014)
		SD rats injected with C6 cells	Orally, 50, 100, and 200 mg/kg for 2 weeks				
*Angelica sinensis* (Oliv.) Diels polysaccharide (ASP)		SH-SY5Y cells	0–500μg/ml, 48 h	AR: Over 10% CV: Under 30% Neuroblastoma	PI3K/AKT Pathway JAK/STAT Pathway	Bax↑; miR-675↓; Bcl-2↓; cleaved Caspase-3, Caspase-9↑; p-PI3K, p-AKT, p-JAK1, p-STAT3↓	(Yang et al., 2018)
*Portulaca oleracea L*. polysaccharide (POL-P3b)		HeLa cells	100 and 200μg/ml, 24 h	AR: About 0.06 Cervical cancer	TLR4/NF-κB Pathway	Bax↑; Bcl-2↓; TLR4, MyD88, TRAF6, AP-1, NF-κB subunit P65↓	(Zhao R. et al., 2017)
*Polygala tenuifolia* Willd. polysaccharide (PTP)		OVCAR-3 cells	0.1–1.6 mg/mL, 24, 48, 72 h	AR: 75.6% Ovarian cancer	NF-κB Pathway Mitochondrial Pathway	Bcl-2↓; Bax, Cytochrome c, Caspase-3, Caspase-9↑; NF-κB↓	(Zhang et al., 2015)
*Angelica sinensis* (Oliv.) Diels polysaccharide (ASP)		T47D cells	12.5, 25, 50, and 100 µg/ml,24 h	Breast cancer	CREB Pathway	CREB↑; Bax↑; Mcl-1↓	(Zhou et al., 2015)
		Mice injected with T47D cells	0.2 mg/kg (i.p.) for 4 weeks				
*Astragalus mongholicus* Bunge polysaccharide (APS)		Hepatocellular carcinoma cells	0.1,0.5,1mg/ml, 3 days	AR: Over 10% Hepatocellular carcinoma	Notch Pathway	Bcl-2↓; Bax↑; Caspase-3 and -8↑; Notch1↓	(Huang et al., 2016)
*Cucurbita pepo* L. (Pumpkin) polysaccharide (PPPF)	Galactose, mannose, glucose, arabinose (2.02:2.05:1.00:0.52)	HepG2 cells	200, 400µg/ml, 48h	AR: 71.5% TIR: 57.56% CV: 28.7% Hepatocellular carcinoma	JAK/STAT Pathways	P-JAK2↓; SHP-1↑	(Shen et al., 2017)
		Female mice injected with HepG2 cells	0.25, 0.5, and 1.0 mg/kg (i.p.) for 18 days				
*Panax ginseng* C.A.Mey. polysaccharide (PGP2a)	Galactose, arabinose, glucose, galacturonic acid (3.7:1.6:0.5:5.4)	HGC-27 cells	100,200,400µg/ml, 48 h	AR: 65.0% CV: Under 50% Gastric cancer	Mitochondrial Pathway Death receptors Pathway Twist/AKR1C2/NF-1 Pathway	Twist, AKR1C2↓; NF1↑; Caspases 3, 9↑; PARP↓; Fas, FasL ↑; Caspase-8↑; Bid↓	(Li C. et al., 2014)
Se-containing polysaccharides from *Pyracantha fortuneana* (Maximowicz) H. L. Li, J. Arnold Arbor. (Se-PFPs)	Xylose, arabinose, fucose, mannose, ribose, rhamnose, glucuronic acid, galacturonic acid, glucose, galactose (29.8:23.2:25.7:4.1:1.8:3.8:2.9:3.2:2.2:3.3)	MDA-MB-231 cells	100, 400μg/ml, 24 h	AR: Over 30% TIR: Nearly 80% Breast cancer	Mitochondrial Pathway CDC25C-CyclinB1/CDC2 pathway	Bax, p53, Puma and Noxa↑; Bcl2↓; Bax/Bcl2↑; Caspases 3/9↑; PARP↓; CDC25C, CDC2 and Cyclin B1↓	(Yuan et al., 2016)
		Female mice injected with MDA-MB-231 cells	Orally, 100,400 mg/kg for 30 days.				
*Angelica sinensis* (Oliv.) Diels polysaccharides (ASP)		U251 cells	0.2 mg/ml, 48 h	Glioma	Mitochondrial Pathway TGF-β Pathway	Bcl-2↓; Bax, cleaved-Caspase-3↑; E-cadherin↑; TGF-β1, p-Smad2, p-Smad3↓	(Yuan et al., 2016)
		Mice injected with U251 cells	50 mg/kg				
*Sparganium stoloniferum* (Buch.-Ham. ex Graebn.) Buch.-Ham. ex Juz. (Sparganii Rhizoma) Polysaccharide (SpaTA)	2-O-grailsine-β-xylose (4→6)-α-glucose (1→4) -β-mannose osamine	ZR-75-1 cells	76.4, 152.8, 305.6, and 611.2 mg/L,24–96h	AR: 40.1% Breast cancer	ERα Pathway	Expression and nuclear translocation of ERα↑; Caspase-3, -8, -9↑; PARP↓	(Wu Y. et al., 2017)

↑ indicates increase/promotion/activation while ↓ indicates inhibition/reduction/inactivation. AR, Apoptosis rate; TIR, Tumor inhibition rate; CV, Cell viability.

## Cancer and Apoptosis

Globally, cancer has been a serious burden on society, and the incidence of cancer is increasing (Zhang H. et al., 2018). In majority cancers, such as lung cancer, breast cancer, liver cancer, stomach cancer, laryngeal cancer, and prostate cancer, genetic mutations are common features (Rosell and Karachaliou, 2015). One of the results of these genetic mutations is the inhibition of apoptosis. Cancer cells escape normal apoptosis, continue to proliferate, interfere with normal organs or tissues, and cause body damage or even death. Therefore, inducing apoptosis has always been one of the exact ways to inhibit cancer. The initiation of apoptosis is the opening or closing of a series of control switches after the corresponding signal stimulates the cell (Milisav et al., 2017). Different signal transduction triggers apoptosis in different ways (Zhang Q. et al., 2019). However, most of the apoptotic pathways eventually work by affecting a group of cysteine proteases called caspases which are revealed as the main executors of the apoptotic pathway due to their role in cleaving the major cellular substrate (Yaacoub et al., 2016). Caspase related to apoptosis is divided into two types, one is the initiator Caspases such as Caspase-2, Caspase-8, Caspase-9, and the other is the effector Caspases including Caspase-3, Caspase-6, Caspase-7 (Kopeina et al., 2018; Sadeghi et al., 2019). The initial Caspase is cleaved and activated under the action of foreign protein signals. The activated initiator cleaves and activates the effector, and finally lyses the cell substrate to cause apoptosis (Ludwig-Galezowska et al., 2011; Van Opdenbosch and Lamkanfi, 2019). Extrinsic death receptor pathway and mitochondrial pathway are two clear pathways that affect apoptosis. They can directly cause Caspase activation to exert a pro-apoptosis role through signaling of Bcl-2 family proteins such as Bcl-2, Bax, Bad, and TNF families such as TNF-R1, Fas, and TRAIL, respectively (Ludwig-Galezowska et al., 2011; Kaufmann et al., 2012). While most other pathways can indirectly affect Caspase proteins, such as MAPK, PI3K/AKT, and NF-κB pathways can regulate apoptosis-related genes and ultimately stimulate mitochondria and death receptors to activate Caspase (Panka et al., 2006; Rohlenova et al., 2016).

## Apoptosis and Plant Polysaccharides

Over the past decades, more than a hundred plant polysaccharides have been discovered, and *in vitro* and *in vivo* studies have shown that most of them have good anticancer activity in a variety of cancers. Lung cancer mice were treated with *Scleromitrion diffusum* (Willd.) R.J.Wang (syn. *Hedyotis diffusa* Willd.) polysaccharide (SDP) which consists of glucose, galactose, mannose ratio of 2.0: 1.0: 1.0. High-dose SDP exhibited tumor inhibition rates comparable to cisplatin-positive drugs (Lin et al., 2019). After treatment with *Citrus × aurantiifolia* (Christm.) Swingle polysaccharide (CAs) in transplanted H22 cells in mice, the tumor suppression rate was as high as 58.85% (Zhao Y. et al., 2017). Moreover, *Polygonatum sibiricum* Redouté polysaccharide had obvious anti-tumor effect on H22 tumor bearing mice (Duan et al., 2014). Similarly, *Saccharina japonica* (J.E. Areschoug) C.E. Lane, C. Mayes, Druehl & G.W. Saunders (syn. *Laminaria japonica*) polysaccharide improved immunomodulatory activity and reduced tumor weight in H22-bearing mice. Simultaneously, the tumor suppression rate can reach 59.67% (Zhu et al., 2016). The effect of *Achyranthes bidentata* Blume polysaccharide (ABPS) on tumor growth depended on its dose. At the doses of 100 mg/kg and 50 mg/kg, ABPS inhibited the growth of mouse Lewis lung cancer by 5.36% and 40.06%, respectively. It was further found that the anti-cancer activity of ABPS was mediated by inducing cell cycle arrest, and that high-dose ABPS stimulated tumor growth was related to NK cell dysfunction, upregulation of IL-6 and TNF-α (Jin et al., 2007).

Clinically, plant polysaccharides also show good curative effects, and are often combined with radiotherapy and chemotherapy. As a result, the toxic and side effects of radiotherapy and chemotherapy can be reduced, while improving the efficacy. *Ginkgo biloba* L. exopolysaccharide (GBEP) capsules were administered orally to 30 patients with gastric cancer. Compared with before treatment, GBEP could reduce the tumor area by 73.4%. The changes of cell ultrastructure suggested that GBEP could induce tumor cell apoptosis and differentiation in patients with gastric cancer (Xu et al., 2003). In clinical trials, *Ginseng* polysaccharide injection was used in combination with chemotherapy to treat advanced malignancies such as lung, stomach and bowel cancer. The results showed that it could reduce the toxic and side effects of chemotherapy on patients, and improve the quality of life of patients, thereby enhancing the compliance of patient with chemotherapy. At the same time, *Ginseng* polysaccharide injection can improve the cellular immune function of patients and enhance the anti-cancer effect (Liu, 2008; Xu, 2015). In a phase II double-blind randomized placebo-controlled trial conducted by *Astragalus* polysaccharides (PG2), advanced head and neck squamous cell carcinoma (HNSCC) was treated with PG2 and chemoradiation. PG2 was found that have the ability to improve the quality of life and adverse reactions that may be related to radiotherapy (Hsieh et al., 2020). Clinical studies have confirmed that *Astragalus* polysaccharides (APS) combined with I125, cisplatin, gemcitabine and platinum, respectively, all can treat non-small cell lung cancer, improve the immune function of patient, reduce the toxicity and side effects of chemo(radio)therapy on patients, and improve the overall quality of life (Qin et al., 2009; Guo et al., 2012; Sun et al., 2014).

On the whole, plant polysaccharides have good effects on the treatment of cancer, and they have excellent clinical value as adjuvant drugs for tumor chemo(radio)therapy. Understanding the therapeutic mechanism of drugs is conducive to a more scientific understanding and development of drugs. Studies have found that polysaccharides can achieve anticancer effects through a variety of mechanisms, such as preventing cancer cell metastasis, enhancing immune activity, and inhibiting division, while the main mechanism is inducing apoptosis of cancer cells (Zong et al., 2012; Khan et al., 2019). Therefore, we will summarize and analyze the specific mechanism of inhibiting cancer by inducing apoptosis of cancer cells by plant polysaccharides.

## Modulation of Apoptosis Pathway by Plant Polysaccharides in Cancer

### Mitochondrial Pathway

The mitochondrial pathway begins with the apoptosis-regulating protein family represented by the Bcl-2 family (Wang et al., 2015). Stimulation of Bcl-2 homology 3 (BH3) proteins such as Bim, Bid, Bad, Puma, Noxa, may transiently interact with Bax or Bak, resulting in the inactivation of Bcl-2 and Bcl-XL and conformational changes of Bax and Bak (Prenek et al., 2017). Activated Bax and Bak can form higher-order homopolymers and stably insert into the outer membrane of mitochondria, which leads to the loss of MMP and promotes the formation of MOMP (Zhou X. et al., 2017; Heinicke et al., 2018). MOMP further promotes the release of cytochrome c into the cytoplasm, which is considered to be a key factor in apoptosis. Once cytochrome c is released, it and Apaf-1 co-activate the initiator Caspase and activate the end effector Caspase to induce apoptosis (Huang et al., 2015). A study in 2015 reported that after the treatment of *Millettia pulchra* (Voigt) Kurz (Yulangshan) polysaccharide (YLSPS), the apoptosis index of 4T1 cells obviously increased, and the primary breast cancer tumors significantly regressed. Related mechanisms have also been revealed, namely that YLSPS could induce mitochondrial-dependent apoptosis by reducing Bcl-2 levels and increasing Bax levels, releasing cytochrome c, and activating caspase-3 (Qin et al., 2019). An *in vivo* investigation showed that *Astragalus membranaceus* Fisch. ex Bunge polysaccharide (APS) inhibited 57.57% of breast cancer tumors by reducing the expression of Bcl-2 protein and up-regulating the expression of Bax, Caspase-9, and Caspase-7. Consequently, this study confirmed the effectiveness of APS in apoptosis regulation (Xie R.-D. et al., 2019). *Glycyrrhiza inflata* Batalin polysaccharide (GIAP1) dose-dependently inhibited the proliferation of SCC-25 cells *via* inducing apoptosis. The relevant mechanism was related to reduce Bax/Bcl-2 ratio, disrupt the MMP, and cause the release of cytochrome c to cytosol. Besides, GIAP1 triggered activation of capase-3 and Caspase-9, as well as the degradation of PARP (Zeng et al., 2015).

In normal cells, ROS can be promptly eliminated by antioxidants such as glutathione, superoxide dismutase and peroxidase, maintained at low levels, and harmless (Dong et al., 2019). However, in tumor cells, ROS production is excessive and cannot be cleared in time, leading to oxidative stress (Dong et al., 2019). If further stimulation is given to increase the production of ROS, it will aggravate oxidative stress and participate in mitochondrial pathway to induce apoptosis (Li et al., 2017a; Cho et al., 2018; Sun et al., 2018). It has been demonstrated that an alkaline polysaccharide (ADAPW) from *Dipsacus asperoides* C.Y.Cheng & T.M.Ai showed apoptosis in human osteosarcoma cell line HOS cells. This was because ADAPW caused a considerable intracellular ROS production, resulting in a remarkable change in MMP (Chen et al., 2013b). APS4, a novel cold-water-soluble polysaccharide was isolated from *Astragalus membranaceus* Fisch. ex Bunge, which had the potential to induce human gastric cancer MGC-803 cell apoptosis. APS4 significantly generated a large amount of ROS and then caused the collapse of MMP, the increase of the pro-apoptotic/anti-apoptotic (Bax/Bcl-2) ratios, ultimately the cleavage of PARP (Yu et al., 2019). Similarly, *Sargassum wightii* Greville ex J.Agardh polysaccharides induced the apoptosis in the breast cancer cells by increasing ROS generation, cleaving mitochondrial membrane and nuclei damage (Vaikundamoorthy et al., 2018).

P53 induces apoptosis mainly through the mitochondrial pathway (Zhang S. et al., 2019). The ability to induce apoptosis may depend on the transcription of the gene and may not be related to transcription (Zhao et al., 2012). Activated p53 moves to the nucleus and regulates the transcription of these pro-apoptotic genes such as Puma, Bax, Bak, Noxa and Bid (Zhao et al., 2012; Yamada and Yoshida, 2019). Additionally, at the beginning of apoptosis, p53 can directly interact with pro-apoptotic factors in mitochondria, activating Bax and/or Bak (Zhao et al., 2012; Yamada and Yoshida, 2019).Se-containing polysaccharides from *Pyracantha fortuneana* (Maxim.) H.L.Li (Se-PFPs) had required potential to combat breast cancer. It has been indicated that Se-PFP could inhibit 80% of breast cancer tumors by inducing apoptosis *via* promoting the expression of p53 and the further increasing Bax, Puma, and Noxa, decreasing Bcl2, and increasing Caspase-3 activity in MDA-MB-231 cells (Yuan et al., 2016). Similarly, it has been reported that peony seed dreg polysaccharides (CASS) triggered apoptosis in cervical cancer Hela cells and this was closely related to the accumulation of P53 by CASS, leading to the release of downstream mitochondrial factors cytochrome c to cytosol, the activation of initiator Caspases- 8 and -9, and subsequent the cleavage of Caspase-3 (Zhang F. et al., 2017). The expression of p53 was significantly increased in the treatment of *Cymbopogon citratus* (DC.) Stapf polysaccharide, which down-regulated the expression of anti-apoptotic factor Bcl-2, up-regulated the pro-apoptotic factor Bax, and induced cell DNA fragmentation. In addition, it could also up-regulate Caspase 3, down-regulate Bcl-2 family genes and promote the release of cytochrome c, and finally successfully achieved the induction of apoptosis to regulate reproductive cancer (Thangam et al., 2014).

Of course, there are many plant polysaccharides that could induce apoptosis of various cancer cells through the mitochondrial pathway similar to the above. The specific mechanism is shown in **Figure 1** and **Table 1**.

### Death Receptor Pathway

Activate death receptors and trigger the family of TNF, including TNF-R1, Fas (also known as CD95 or Apo-1 TNFRSF6), TRAIL receptors (TRAIL-R1, TRAIL-R2, also known as DR4 and DR5), DR3 and DR6, are also common apoptosis pathways (Zhou X. et al., 2017). These receptors are stimulated and involved by the corresponding ligand, leading to the aggregation of receptors, the recruitment of FADD, and subsequent accumulation of proCaspase-8, which in turn activates Caspase-8 (Huang et al., 2018). Activated Caspase-8 can directly activate Caspase-3, -7 and play a pro-apoptotic effect (Liang et al., 2018). Caspase-8 can also induce MOMP and trigger the release of cytochrome c by cleaving Bid, initiating the effect of Caspase activation and apoptosis (Zhou X. et al., 2017). **Figure 2** depicts the pro-apoptotic activity of plant polysaccharides *via* death receptor pathway. Several plant polysaccharides have been found to affect death receptors. The anti-laryngeal cancer activity of *Boschniakia rossica* (Cham. & Schltdl.) B.Fedtsch. polysaccharide (BRP) was the result of its induction of apoptosis in Hep2 cells through death receptor-mediated pathway. The specific mechanism is that BRP promoted nitric oxide (NO) production and increased the secretion of TNF (Yao et al., 2017), and also increased the expression of death receptor DR5 and promoted pro-Caspase-3, pro-Caspase-8, pro-Caspase-9 and Bax cleavage, and reduced Bcl-2 (Wang et al., 2014). Qu et al. found that a polysaccharide, TFPB1, from the flower buds of *Tussilago farfara* L. exerted a pro-apoptotic effect in human lung cancer A549 cells, while up-regulating the expression of Fas, FasL, and Bax, and down-regulating the expression of Bcl-2 is one of the reasons (Qu et al., 2018). Death receptor-mediated extrinsic apoptotic pathway was involved in the *Panax ginseng* C.A.Mey. polysaccharide (PGP2a)-induced apoptosis in HGC-27 cells, which was manifested in that PGP2a increased significantly the expression of Fas and FasL, further promoted the cleavage of pro-Caspase-8, and activated Caspase-9 and Caspase-3 (Li C. et al., 2014). *Ginger* polysaccharide (GP), which consists of L-rhamnose, D-arabinose, D-mannose, D-glucose, and D-galactose in a molar ratio of 3.64: 5.37: 3.04: 61.03: 26.91, has been shown that have good effects in the induction of hepatocellular carcinoma HepG2 cells apoptosis. This may be related to up-regulate the expression of Bax, Fas, FasL, Caspase-3, p21, and p53, and down-regulate the expression of Bcl-2 (Wang et al., 2019). In lung cancer A549 cells, *Gracilariopsis lemaneiformis* (Bory de Saint-Vincent) E.Y.Dawson, Acleto & Foldvik polysaccharide (PGL) induced apoptosis by activating genes involved in the death receptor apoptotic pathway and that the Fas/FasL signaling pathway might play a critical role (Kang et al., 2017). Interestingly, *Carthamus tinctorius* L. polysaccharide (SPS) injection also inhibited 80% of lung cancer tumor growth in mice, probably due to its positive effect on TNF-α and IL-6 expression (Li J. Y. et al., 2016). Combination medication is often an effective way to treat disease. Chen et al. studied the effect of combination of *Lycium barbarum* L. polysaccharide (LBP) and TRAIL on MLL rearranged leukemic cells. The results showed that a significant increase in the expression level of DR5 receptor, Caspase-8, Caspase-3, Caspase-9, and Bad, indicating that LBP and TRAIL can further increase the sensitivity of MLL-ALL cell lines to TRAIL-induced apoptosis by increasing the expression level of DR5 receptors on cells (Chen C. et al., 2019).

### The Regulation of Signal Conduction

Apoptosis can be induced not only directly through the mitochondrial pathway and death receptor pathway, but also by affecting their upstream signal transduction (Wei et al., 2011). Plant polysaccharides have been found that they could affect some upstream signal transduction such as MAPK Pathway, PI3K/AKT Pathway, NF-κB Pathway, as shown in **Figure 3** and **Table 1**.

#### MAPK Pathway

Many of the signaling pathways that regulate cancer cell apoptosis are members of the MAPKs family (Papa et al., 2019). Extracellular signal-regulated kinase (ERK), c-Jun N-terminal kinase (JNK), and p38 kinase are three well-defined MAPK subfamilies in mammals (Petrache et al., 1999). JNK promotes apoptosis through two different mechanisms. The translocation of activated JNK to the nucleus increases the expression of pro-apoptotic genes by activating c-Jun/AP1 dependent mechanisms (Dhanasekaran and Reddy, 2008). On the other hand, the activated JNK translocates to mitochondria and phosphorylates BH3 protein, thereby antagonizing Bcl-2 and Bcl-XL, and finally exert anti-apoptotic activity (Iurlaro and Muñoz-Pinedo, 2016). ERK is one of the anti-apoptotic members. ERK is usually deregulated in tumors due to mutations in Ras or B-Raf. It mainly exerts a anti-apoptotic effect by promoting the activity of anti-apoptotic proteins such as Bcl-2, Bcl-XL, Mcl-1, IAP, and inhibiting apoptotic proteins such as Bad and Bim (Westhoff and Fulda, 2009; Cagnol and Chambard, 2010).While p38 MAPK-dependent apoptosis is mediated by STAT1, CHOP, FAK, SMAD, cytochrome c, NF-κB, PTEN, and p53-mediated downstream events (Yokota and Wang, 2016). SCP, a polysaccharide from *Schisandra chinensis* (Turcz.) Baill., showed high cytotoxic potential in Caki-1 cells by inducing apoptosis, which was related to inhibit ERK1/2 phosphorylation and further induce apoptosis in the mitochondrial pathway (Liu et al., 2014). *Aconitum coreanum* (H.Lév.) Rapaics polysaccharide (CACP) could significantly inhibit the growth of hepatocellular carcinoma cells, and its tumor suppression rate could reach 45.9%, which is the result of CACP treatment activating the p-p38 MARK-dependent apoptosis pathway (Liang et al., 2015). It has been reported *G. lemaneiformis* polysaccharide-Nano-selenium particles (GLP-SeNPs) had high selectivity between normal cells and cancer cells, and could effectively and safely fight cancer. Its anticancer effect was mainly dependent on the MAPKs pathway. GLP-SeNPs were capable of upregulating the phosphorylation of pro-apoptotic kinases p38 and JNK in a dose-dependent trend, however, remarkably repressing the phosphorylation of antiapoptotic kinases AKT and ERK (Jiang et al., 2014). Cisplatin is one of common chemotherapy drugs that can kill cancer cells by mediating apoptosis. Interestingly, APS could increase the sensitivity of ovarian cancer SKOV3 cells to cisplatin, which may be related to APS activating JNK1/2 signalling pathway (Li C. et al., 2018).

#### PI3K/AKT Pathway

PI3K/AKT is involved in regulating cell proliferation, apoptosis and cell cycle, and is an anti-apoptotic pathway worth exploring (Franke et al., 2003; Liu et al., 2019). In tumor cells the PI3K/AKT signaling pathway is mostly activated (Xie et al., 2017). Activated AKT directly phosphorylates Ser136 of Bad, which binds to the 14-3-3 protein, promotes Bcl-2/Bcl-XL expression, thereby inhibiting apoptosis through the mitochondrial pathway (Chang et al., 2003; Arlt et al., 2013). In addition, AKT can inhibit apoptosis by activating NF-κB (Chang et al., 2003). Blocking this pathway can effectively promote cancer cell apoptosis to exert anti-cancer effect. SPS2p is a *Scutellaria barbata* D.Don polysaccharide that could inhibit the activation of the PI3K/AKT signaling pathway in colon cancer cells. This was demonstrated by the fact that SPS2p could promote HT29 cell apoptosis *via* down-regulating the ratio of p-AKT/AKT, and the expression of Bcl-2 (Sun et al., 2017). It was found that *Aster tataricus* L.f. polysaccharide (ATP-II) induced apoptosis in glioma cells by reducing the PI3K/AKT signaling pathway. Specifically, ATP-II down-regulated p-AKT protein expression and regulated the ratio of AKT-mediated pro-apoptotic and anti-apoptotic protein Bax/Bcl-2 (Du et al., 2014). Chen et al. studied the antitumor effect of *G. inflata* polysaccharide (GPSa) on human hepatocellular carcinoma cells and its mechanism, and it was found that GPSa had good anticancer activity *in vitro* and *in vivo*. The possible mechanism was that it promoted the apoptosis of H22 cells by influencing the P53/PI3K/AKT pathway (Chen et al., 2013a). In addition, Kwon and Nam found that *Capsosiphon fulvescens* (C.Agardh) Setchell & N.L.Gardner polysaccharide (Cf-PS) stimulated AGS cells significantly decreased Bcl-2 expression, activated Caspase-3, inactivated the PI3K/AKT pathway, and showed anti-gastric cancer activity (Kwon and Nam, 2007). In 2016, Li et al. reported that SPS inhibited tumor growth by reducing the expression of AKT, p-AKT, and PI3K and increasing mRNA levels of Bax and Caspase-3 (Li J. Y. et al., 2016). In hepatocellular carcinoma HepG2 cells, *Cornus officinalis* Siebold & Zucc. polysaccharide could promote the expression of apoptosis-related proteins Bax and Caspase-3, and inhibit the expression of Bcl-2 and PI3K/AKT pathway-related proteins, thus showing pro-apoptotic activity (Li and Sun, 2019). In the same way, CACP could promote apoptosis by inactivating the P13K/AKT signaling pathway and inhibit H22 cell growth *in vitro* and *in vivo* (Liang et al., 2015).

#### NF-κB Pathway

NF-κB is one of the key factors controlling the anti-apoptotic response (Namba et al., 2007). In the cytoplasm, NF-κB (p50/p65) and lkB combine to form a trimer, and p50/p65 is unable to undergo nuclear translocation in the resting state of cell (Yu et al., 2017). When cells are stimulated by specific external signals, IKKβ subunit of IKK is activated by phosphorylation, which in turn causes phosphorylation of Ser32 and Ser36 sites of IkBα (Hayden and Ghosh, 2004). Phosphorylated IkBα is then degraded to p50/p65 for nuclear translocation (Hayden and Ghosh, 2004; Oeckinghaus and Ghosh, 2009). Translocated NF-κB induces expression of anti-apoptotic genes, such as Bcl-Xl and Bcl-2, leading to enhance survival while avoiding apoptosis of most cells (Ward et al., 2004; Namba et al., 2007; Nguyen et al., 2014). Multiple chemotherapeutics play an anti-cancer role by blocking this pathway (Zhang K. et al., 2019). In OVCAR-3 cells, in addition to significantly affect the mitochondrial pathway, *Polygala tenuifolia* Willd. polysaccharide (PTP) treatment could reduce the expression of NF-κB, thereby triggering cell death signals in the programmed external pathway of tumor cells (Zhang et al., 2015). A similar mechanism was found in *Hordeum vulgare L*. polysaccharide (BP-1) to stimulate human colon cancer cells (HT-29). BP-1 could inhibit the transfer of NF-κB from the cytoplasm to the nucleus. This further affected apoptosis-related proteins, such as Bcl-2, promoted the release of cytochrome c from mitochondria to the cytoplasm, and the activation of Caspase-8 and Caspase-9 to induce apoptosis (Cheng et al., 2016). Furthermore, *Portulaca oleracea* L. Polysaccharide (POL-P3b) could inhibit the expression of TLR4, MyD88, TRAF6, AP-1, and NF-κB subunit P65 in HeLa cells, thereby inhibiting the TLR4/NF-κB pathway to induce Cervical cancer cells apoptosis (Zhao R. et al., 2017).

### Other Pathways

Apoptosis is regulated by multiple targets and multiple pathways. In addition to the above mechanisms, plant polysaccharides can affect many other pathways to induce apoptosis. CREB is a major inductor of apoptosis activation, ASP could regulate the activation of Bcl-2 through CREB signaling pathway to induce apoptosis and to produce a profound antitumor effect on T47D cells (Zhou et al., 2015). Abnormal activation of STAT3 is necessary for the survival of many human cancer cells, and inhibition of JAK2/STAT3 pathway by inhibitors or siRNAs can reduce cell survival and induce apoptosis (Shu et al., 2011). Shen et al. reported that Pumpkin polysaccharide (PPPF)-induced apoptosis in HepG2 cells may be mediated by the JAK2/STAT3 signal transduction pathway (Shen et al., 2017). The expression, abundance, and activity of ERα were increased in most of breast cancers (Lundqvist et al., 2018). *Sparganium stoloniferum* (Buch.-Ham. ex Graebn.) Buch.-Ham. ex Juz. polysaccharide (SpaTA) could regulate the expression of ERα and activate its genomic functions by inducing the downstream estrogen pathway, and then further activated the Caspase cascade to exert apoptosis in an ERα-dependent manner (Wu Y. et al., 2017). Twist and its downstream AKR1C2 and NF1 genes can induce tumor progression, cell proliferation and tumorigenesis in HGC-27 cells. It has been found that PGP2a-induced gastric cancer HGC-27 cell apoptosis may be mediated by regulating the expression of Twist and its downstream genes (Li C. et al., 2014). G2/M phase arrest is closely related to early cell apoptosis. SPS induced lung cancer cells cycle arrest in the G2/M phase by reducing the expression of CDC25B and cyclin B1 (Li J. Y. et al., 2016).

## The Absorption of Plant Polysaccharides *In Vivo*

Drugs must first enter the body in order to work, so the absorption pathways and bioavailability of plant polysaccharides should be discussed. Studies have found that polysaccharides may be absorbed by the intestine after oral administration. As early as 2014, Liao established a Caco-2 cell transport model and found that both *Gastrodia* polysaccharide and *Grifola frondosa* (Dicks). Gray polysaccharides could successfully transmembrane, and the polysaccharides remained basically unchanged without degradation. Further studies on its absorption pathway revealed that the uptake of two polysaccharides in the small intestinal cells is the clathrin heavy chain endocytosis pathway (Liao, 2014). A similar study was discovered in 2017. After oral administration of ASP, ASP could be absorbed through the large cell-drinking route and clathrin and small concave (or lipid raft) -related endocytosis, and enter the blood circulation to exert effect (Wang et al., 2017). While the absorption of *Gynochthodes officinalis* (F.C.How) Razafim. & B.Bremer (syn. *Morinda officinalis* F.C.How) polysaccharide in the small intestine was found to be accomplished through passive diffusion (Chen et al., 2012). However, in general, the oral absorption rate of polysaccharides is negatively affected by its molecular weight, and the bioavailability of most plant polysaccharides is low (Yi et al., 2014). To improve the bioavailability and pharmacological effects of bioactive polysaccharides, various particle delivery systems have been developed, such as liposomes, nanovesicles, nanoemulsions, and micelles (Li et al., 2017b; Ruttala et al., 2017). *Morus alba* L. (Fructus Mori) polysaccharides (MFP) were spheroidized by anti-solvent precipitation method. The oral bioavailability of MFP and its particles (MFP-NP1, MFP-NP2, MFP-NP3 and the size of their spheroidization is MFP-NP1> MFP-NP2> MFP-NP3) were evaluated by measuring the polysaccharide content in the plasma of mice. After 4 h of administration, the concentrations of MFP, MFP-NP1, MFP-NP2 and MFP-NP3 were 0.28, 0.58, 0.69, and 0.97μg/ml, respectively. The relative bioavailability of MFP- NP1, MFP- NP2, and MFP- NP3 are 4.81, 6.33, and 8.54 times, respectively, which are inversely related to their size (Chen and Fu, 2019). In addition, encapsulation of nanoencapsulated polysaccharides extracted from *Taiwanofungus camphoratus* (M. Zang & C.H. Su) Sheng H. Wu, Z.H. Yu, Y.C. Dai & C.H. Su (syn. *Antrodia camphorata* (M. Zang & C.H. Su) Sheng H. Wu, Ryvarden & T.T. Chang), enhanced its biological activity (Chang et al., 2015). The encapsulated nanoemulsion of *Anthophycus longifolius* (Turner) Kützing (syn. *Sargassum longifolium* (Turner) C.Agardh) polysaccharide was pretreated and sustainedly released *in vitro*, indicating an increase in its bioavailability (Shofia et al., 2018). *Ophiopogon japonicus* (Thunb.) Ker Gawl. polysaccharides (ROPs)-loaded liposomes showed more efficient and stronger antigen-specific immune responses than free ROPs (Fan et al., 2016). Interestingly, recent studies have shown that certain polysaccharides can inhibit tumors in the host. However, they cannot rely on absorption into the body to work due to their large molecular weight, but directly affect the gut microbiota. The gut microbiota has a symbiotic relationship with the host, plays a decisive role in host nutrition, immunity and metabolism, and it can regulate the effect of cancer treatment (Chen D. et al., 2019). For example, in a colorectal cancer mouse model, licorice and jujube polysaccharides positively regulated the intestinal microbial flora and affected certain metabolic pathways that are beneficial to the health of the host to exert antitumor effects (Zhang X. et al., 2018; Ji et al., 2020). After oral administration *Dendrobium officinale* Kimura & Migo (syn. *Dendrobium huoshanense* Kimura & Migo) polysaccharide, it could significantly change the physiological state of the intestine, enhance the function of the intestinal physiology, biochemistry and the intestinal immunological barrier, and regulate the composition of microbial population and microbial metabolism (Xie S. et al., 2019). In a study in 2019, *D. officinale* polysaccharide (DOP) was shown to have anti-breast cancer effects. But it was indigestible and non-absorbable, and eventually it was discovered that DOP exerted the effect by regulating the components of the intestinal flora related to anti-breast cancer (Li L. et al., 2019).

## The Toxicity Analysis of Plant Polysaccharides

In the clinic, the treatment of cancer is a long-term process, so the study of drug toxicity is also necessary. The application of most plant polysaccharides to cancer shows no toxicity. Lung cancer was treated with SDP for 15 days, and all nude mice were observed to show no signs of death or toxicity. Further research found that SDP could induce A549 cells apoptosis, but had no significant effect on normal human fibroblasts WI38 (Lin et al., 2019). In addition, no mice died and no significant reduction in body weight was observed during 3 weeks of CAs-treated liver cancer, indicating that CAs have no significant toxicity to host animals (Zhao Y. et al., 2017). In one study, licorice polysaccharides have been shown to increase immune organ weight and index, activate immune cells, thereby inhibiting tumor growth, and had no toxic manifestations in CT 26 tumor-bearing mice (Ayeka et al., 2017). Chen et al. carried out a toxicity study of YLSP in male and female rats, and found that a single oral administration of 24 g/kg YLSP in the acute toxicity study did not cause toxicological symptoms or cause death. Also, in the chronic toxicity study, intragastric administration was performed for 26 weeks, followed by a 3-week recovery period, and no death or significant clinical symptoms were observed. In addition, no toxicity was found in measurements of body weight, food consumption, hematology, clinical biochemistry, organ weight (Chen et al., 2016). Male and female rats were given tamarind polysaccharide for 28 days without death and no pathological findings (Heimbach et al., 2013). In the toxicity test of *Codonopsis pilosula* (Franch.) Nannf. polysaccharide oral solution administered to rats, there were no significant changes in the general condition, behavior, hematology, blood biochemistry, and main organ pathology of rats in each group after 4 weeks of administration and two weeks after withdrawal (Hou et al., 2016). APS had no obvious toxicity in 30-day long-term toxicity test in rats. The growth and development of rats, viscera-body ratio, and other indicators did not show significant effects, and gross anatomy and histological observation showed no abnormal pathological changes (Liu and Xiao, 2011). In addition, plant polysaccharides from *Aster tataricus*, Panax ginseng, *Angelica sinensis*, *Aralia elata* (Miq). Seem., pumpkin and most others have no obvious toxic and side effects on normal cells or nude mice during the treatment process (Du et al., 2014; Li C. et al., 2014; Zhou et al., 2015; Liu et al., 2016b; Sun et al., 2016; Shen et al., 2017).

## Conclusions and Perspectives

Multi-component plants and their extracts are often used to successfully treat various diseases, including suppressing cancer. Polysaccharides are active ingredients widely present in a large number of plants, and they can induce apoptosis through multiple targets and multiple pathways in the treatment of cancer. Therefore, in this review, we evaluated related articles that revealed how fifty plant polysaccharides can induce apoptosis and play a role in improving cancer cells in a multi-target, multi-path way. Mitochondrial-mediated pathway, death receptor pathway, and their upstream signal transduction such as MAPK pathway, PI3K/AKT pathway and NF-κB pathway are important pathways that affect apoptosis. As shown in **Figures 1**–**3**, plant polysaccharides can effectively play a pro-apoptotic effect and effectively improve cancer through these pathways. However, different plant polysaccharides can perform different apoptotic pathways, and different plant polysaccharides have different effects and mechanisms for different types of cancer, as shown in **Table 1**. And the high cancer cell apoptosis rate, cancer inhibition rate, and low cancer cell vitality reflect the superior anti-cancer effect of plant polysaccharides. The activity of polysaccharides is expressed in different units, which is difficult to compare with traditional anticancer drugs. However, at normal dosages, some plant polysaccharides had the same strength in inhibiting cancer as radiotherapy and chemotherapy. In addition, polysaccharide activity is closely related to structural characteristics, but plant polysaccharides are mostly mixtures in nature with complex structures and various types. Isolation, purification and structure determination of polysaccharides are difficult. Therefore, as shown in **Table 1**, the structural characteristics of most of the reported active polysaccharides are still unclear, and structure-activity relationships cannot be studied in detail. Nevertheless, the structural diversity and activity of polysaccharides depend to a large extent on their plant source. We have concluded that most of polysaccharides that induce apoptosis and produce anticancer activity come from these plants which are both functional foods and medical drugs, this is what we often call drug and food homology. In daily life, these plants are often widely used to supplement nutrition, and no serious physical injuries have been found. Therefore, in the research of anticancer drugs, we can pay close attention to these plants or their components that are homologous to medicinal and food products, which may have both strong biological activity and excellent safety.

In addition, we found that after oral administration some plant polysaccharides can be absorbed in the small intestine by passive diffusion, endocytosis, and so on. However, due to the large molecular weight of most polysaccharides, their bioavailability is still generally low. Using nanometer and liposome for encapsulation was found to improve their bioavailability and bioactivity. Subsequently, the effect of plant polysaccharides on the intestinal microflora provides us with new ideas. This can reveal why plant polysaccharides are not well absorbed but can inhibit tumor growth *in vivo*. So, in the subsequent research on the anticancer mechanism of plant polysaccharides, we can take influencing the gut microbiota as an important direction.

The ultimate purpose of the drug is to serve the clinic. Studies have shown that most plant polysaccharides have obvious anti-cancer effects and are non-toxic to normal organisms, ensuring clinical effectiveness and safety. However, the existing clinical research on plant polysaccharides is limited, and most polysaccharides have not been studied in human-specific clinical trials. One possible reason is that most plant polysaccharides are currently focused on anti-cancer mechanism research, but no overall animal experiments have been conducted to determine the efficacy of polysaccharides *in vivo*. Consequently, it is hoped that in the future research, the anticancer effect of plant polysaccharides *in vivo* can be comprehensively researched and entered into the clinical research stage as soon as possible.

## Author Contributions

Q-WH is the corresponding author on the study. Q-XG and JW are first authors and responsible for collecting materials and writing the paper. JH, G-HL, H-JX, and C-YP helped in organizing the information and edited the article pictures. All authors read and approved the final manuscript.

## Funding

This work is financially supported by the Key R & D projects (Code:2018SZ0077) and the applied basic research project(Code:2018JY0032), both of Sichuan Science and Technology Department, by the Chengdu Science and Technology Bureau Technology R & D Project (Code: 2015-HM01-00401-SF)

## Conflict of Interest

The authors declare that the research was conducted in the absence of any commercial or financial relationships that could be construed as a potential conflict of interest.

## Abbreviations

AKR1C2, aldo-keto reductase family 1 member C2; AKT, protein kinase b; AP-1, Activator Protein-1; Bad, Bcl-2-antagonist of cell death; Bak, BCL- 2 antagonist or killer; Bax, BCL-2-associated X protein; Bcl-2, B cell lymphoma 2; CDC, cell division cycle; Cdk, cyclin dependent kinases; COX- 2, prostaglandin-endoperoxide synthase 2; CREB, the Cyclic AMP response element binding protein; ERα, estrogen receptor alpha; FN, fibronectin; GSK-3β, glycogen synthase kinase 3β; IGF-IR, insulin-like growth factor-I receptor; IKK, inhibitor of NF-κB kinase subunit; IL-6, interleukin 6; i.p., intraperitoneal injection; IκBα, NF-κB inhibitor alpha; JAK, Janus kinase; MAPK, mitogen activated protein kinase; Mcl-1, Myeloid cell leukemia-1; MMP, mitochondrial membrane potential; MMP-9, matrix metalloproteinase-9; MOMP, mitochondrial outer membrane permeabilization; MyD88, myeloid differentiation primary response gene 88; NF-κB, nuclear factor kappa B; Noxa, Apaf-1,apoptotic protease activating factor 1; PARP, poly (ADP-ribose) polymerase; PI3K, phosphoinositol-3-kinase; Puma, p53-upregulated modulator of apoptosis; PTTG1, pituitary tumor transforming gene 1; p-, phosphorylation; P38, p38 MAP kinase; ROS, reactive oxygen species; STAT, signal transducer and activator of transcription; TGF, transforming growth factor; TIMP, tissue inhibitors of metalloproteinase; TLR4, toll-like receptor 4; TNF, tumor necrosis factor; TNF-R1, tumor necrosis factor receptor 1; TRAIL, TNF-related apoptosis-inducing ligand; VEGF, vascular endothelial growth factor.
